# Supplementary material for: Heat adaptation of phage T7 under an extended genetic code
Source: Virus Evol. 2021 Dec 1;7(2):veab100. doi: 10.1093/ve/veab100 (PMC8923235; doi:10.1093/ve/veab100)
Supplement: veab100_Supp [file veab100_supp.zip › Sequencing_Analysis.zip!/Sequencing_Analysis/S2/evidence/JC_SIDE_2_55.html]

BRESEQ :: Evidence


| New junction evidence | | | | | | | | | | | |
| --- | --- | --- | --- | --- | --- | --- | --- | --- | --- | --- | --- |
|  | | seq id | position | reads (cov) | reads (cov) | score | skew | freq | annotation | gene | product |
| \* | ? | NC\_001604.1 | = 8335 | 5558 (1.050) | 25 (0.000) | 22/584 | 44.4 | 0.45% | intergenic (–/–) | *–/–* | –/– |
|  | ? | NC\_001604.1 | 8939 = | 5643 (1.100) |  |  |  |  | intergenic (–/–) | *–/–* | –/– |
| Rejected: Coverage evenness skew score above cutoff. | | | | | | | | | | | |
| Rejected: Frequency below/above cutoff threshold. | | | | | | | | | | | |

Only 100 of 5757 total aligned reads displayed.  

`TAAAGTACAGACTACGTGCTGGTAAGAAGTCAGAGTTAGCGTACTTAGAGAAAGACCTAGCGAAAGCAGACTTCTATAAAGAACTCTTTGAGAAACATAAGGATAAATGTTATGCATAACTTCAAGTCAACCCCACCTGCCGACAGCCTATCTGATGACTTCACATCTTGCTCAGAGTGGTGCCGAAAGATGTGGGAAGAGACATTCGACGATGCGTACATCAAGCTGTATGAAC‑TTTGGAAATCGAGAGGTCAATGACTATGTCAAACGTAAATACAGGTTCACTTAGTGTGGACAATAAGAAGTTTTGGGCTACCGTAGAGTCCTCGGAGCATTCCTTCGAGGTTCCAATCTACGCTGAGACCCTAGACGAAGCTCTGGAGTTAGCCGAATGGCAATACGTTCCGGCTGGCTTTGAGGTTACTCGTGTGCGTCCTTGTGTAGCACCGAAGTAATACGACTCACTATTAGGGAAGACTCCCTCTGAGAAACCAAACGAAACCTAAAGGAGATTAACATTATGGCTAAGAAGATTTTCACCTCTGCGCTGGGTACCGCTGAACCTTACGCTTACATCGCCAAGCCGGACTACGGCAACGAAG  >  NC_001604.1/8638‑9239`  

                                                                                                                                                                                                                                                                                                              `|`                                                                                                                                                                                                                                                                                                              
    `gTACAGACTACGTGCTGGTAAGAAGTCAGAGTTAGCGTACTTAGAGAAAGACCTAGCGAAAGCAGACTTCTATAAAGAACTCTTTGAGAAACATAAGGATAAATGTTATGCATAACTTCAAGTCAACCCCACCTGCCGACAGCCTATCTGATGACTTCACATCTTGCTCAGAGTTGTGCCGAAAGATGTGGGGAGAGACATTCGACGATGCGTACATCAAGCTGTATGAAC‑TTTGGAAATCGAGAGGTCAATGACTATGTCAAACGTAAATACAGGTTCACTTAGTGTGGACAATaagaag                                                                                                                                                                                                                                                                                                           >  1:788702/1‑301 (MQ=255)`  
                 `gCTGGTAAGAAGTCAGAGTTAGCGTACTTAGAGAAAGACCTAGCGAAAGCAGACTTCTATAAAGAACTCTTTGAGAAACATAAGGATAAATGTTATGCATAACTTCAAGTCAACCCCACCTGCCGACAGCCTATCTGATGACTTCACATCTTGCTCAGAGTGGTGCCGAAAGATGTGGGAAGAGACATTCGACGATGCGTACATCAAGCTGTATGAAC‑TTTGGAAATCGAGAGGTCAATGACTATGTCAAACGTAAATACAGGTTCACTTAGTGTGGACAATAAGAAGTTTTGGGCTACCg                                                                                                                                                                                                                                                                                              <  1:359086/301‑1 (MQ=255)`  
                  `cTGGTAAGAAGTCAGAGTTAGCGTACTTAGAGAAAGACCTAGCGAAAGCAGACTTCTATAAAGAACTCTTTGAGAAACATAAGGATAAATGTTATGCATAACTTCAAGTCAACCCCACCTGCCGACAGCCTATCTGATGACTTCACATCTTGCTCAGAGTGGTGCCGAAAGATGTGGGAAGAGACATTCGACGATGCGTACATCAAGCTGTATGAAC‑TTTGGAAATCGAGAGGTCAATGACTATGTCAAACGTAAATACAGGTTCACTTAGTGTGGACAATAAGAAGTTTTGGGCTACCGt                                                                                                                                                                                                                                                                                             <  1:18377/301‑1 (MQ=255)`  
                    `ggTAAGAAGTCAGAGTTAGCGTACTTAGAGAAAGACCTAGCGAAAGCAGACTTCTATAAAGAACTCTTTGAGAAACATAAGGATAAATGTTATGCATAACTTCAAGTCAACCCCACCTGCCGACAGCCTATCTGATGACTTCACATCTTGCTCAGAGTGGTGCCGAAAGATGTGGGAAGAGACATTCGACGATGCGTACATCAAGCTGTATGAAC‑TTTGGAAATCGAGAGGTCAATGACTATGTCAAACGTAAATACAGGTTCACTTAGTGTGGACAATAAGAAGTTTTGGGCTACCGTag                                                                                                                                                                                                                                                                                           <  1:654514/301‑1 (MQ=255)`  
                      `tAAGAAGTCAGAGTTAGCGTACTTAGAGAAAGACCTAGCGAAAGCAGACTTCTATAAAGAACTCTTTGAGAAACATAAGGATAAATGTTATGCATAACTTCAAGTCAACCCCACCTGCCGACAGCCTATCTGATGACTTCACATCTTGCTCAGAGTGGTGCCGAAAGATGTGGGAAGAGACATTCGACGATGCGTACATCAAGCTGTATGAAC‑TTTGGAAATCGAGAGGTCAATGACTATGTCAAACGTAAATACAGGTTCACTTAGTGTGGACAATAAGAAGTTTTGGGCTACCGTagag                                                                                                                                                                                                                                                                                         <  1:116517/301‑1 (MQ=255)`  
                      `tAAGAAGTCAGAGTTAGCGTACTTAGAGAAAGACCTAGCGAAAGCAGACTTCTATAAAGAACTCTTTGAGAAACATAAGGATAAATGTTATGCATAACTTCAAGTCAACCCCACCTGCCGACAGCCTATCTGATGACTTCACATCTTGCTCAGAGTGGTGCCGAAAGATGTGGGAAGAGACATTCGACGATGCGCACATCAAGCTGTATGAAC‑TTTGGAAATCGAGAGGTCAATGACTATGTCAAACGTAAATACAGGTTCACTTAGTGTGGACAATAAGAAGTTTTGGGCTACCGTagag                                                                                                                                                                                                                                                                                         <  1:761057/301‑1 (MQ=255)`  
                      `aaagaagTCTGAGTTAGCGTACTTAGAGAAAGACCTAGCGAAAGCAGACTTCTATAAAGAACTCTTTGAGAAACATAAGGATAAATGTTATGCATAACTTCAAGTCAACCCAAACTGCCGACAGCCTATCTGATGACTTCACATCTTGCTCAGAGTGGTGCCGAAAGATGTGGTAAGAGACATTCGACGATGCGTACATCAAGCTGTATGAAC‑TTTGGAAATCGAGAGGTCAATGACTATGTCAAACGTAAATACAGGTTCACTTAGTGTGGACAATAAGAAGTTTTGGGCTACCGTagag                                                                                                                                                                                                                                                                                         <  1:502049/300‑1 (MQ=255)`  
                      `aaagaagTCAGAGTTAGCGTACTTAGAGAAAGACCTAGCGAAAGCAGACTTCTATAAAGAACTCGTTGAGAAACATAAGGATAAATGTTATGCATAACTTCAAGTCAACCCCACCTGCCGACAGCCTATCTGATGACTTCACATCTTGCTCAGAGTGGTGCCGAAAGATGTGGGAAGAGACATTCGACGATGCGTACATCAAGCTGTATGAAC‑TTTGGAAATCGAGAGGTCAATGACTATGTCAAACGTAAATACAGGTTCACTTAGTGTGGACAATAAGAAGTTTTGGGCTACCGTagag                                                                                                                                                                                                                                                                                         <  1:388429/300‑1 (MQ=255)`  
                            `gTCAGAGTTAGCGTACTTAGAGAAAGACCTAGCGAAAGCAGACTTCTATAAAGAACTCTTTGAGAAACATAAGGATAAATGTTATGCATAACTTCAAGTCAACCCCACCTGCCGACAGCCTATCTGATGACTTCACATCTTGCTCAGAGTGGTGCCGAAAGATGTGGGAAGAGACATTCGACGATGTGTACATCAAGCTGTATGAACTTTTGGAAATCGAGAGGTCAATGACTATGTCAAACGTAAATATAGGTTCACTTAGTGTGGACAACAAGAAGTTTTGGGCTACCGTAGAGTCCTc                                                                                                                                                                                                                                                                                    >  1:753707/1‑301 (MQ=255)`  
                             `tCAGAGTTAGCGTACTTAGAGAAAGACCTAGCGAAAGCAGACTTCTATAAAGAACTCTTTGAGAAACATAAGGATAAATGTTATGCATAACTTCAAGTCAACCCCACCTGCCGACAGCCTATCTGATGACTTCACATCTTGCTCAGAGTGGTGCCGAAAGATGTGGGAAGAGACATTCGACGATGCGTACATCAAGCTGTATGAAC‑TTTGGAAATCGAGAGGTCAATGACTATGTCAAACGTAAATACAGGTTCACTTAGTGTGGACAATAAGAAGTTTTGGGCTACCGTAGAGTCCTCgg                                                                                                                                                                                                                                                                                  <  1:729285/301‑1 (MQ=255)`  
                                `gtgtataagagacaggagaAAGACCTAGCGAAAGCAGACTTCTATAAAGAACTCTTTGAGAAACATAAGGATAAATGTTATGCATAACTTCAAGTCAACCCCACCTGCCGACAGCCTATCTGATGACTTCACATCTTGCTCAGAGTGGTGCCGAAAGATGTGGGAAGAGACATTCGACGATGCGTACATCAAGCTGTATGAAC‑TTTGGAAATCGAGAGGTCAATGACTATGTCAAACGTAAATACAGGTTCACTTAGTGTGGACAATAAGAAGTTTTGGGCTACCGTAGAGTCCTCGGAGc                                                                                                                                                                                                                                                                               <  1:381942/286‑1 (MQ=255)`  
                                `gagTTAGCGTACTTAGAGAAAGACCTAGCGAAAGCAGACTTCTATAAAGAACTCTTTGAGAAACATAAGGATAAATGTTATGCATAACTTCAAGTCAACCCCACCTGCCGACAGCCTATCTGATGACTTCACATCTTGCTCAGAGTGGTGCCGAAAGATGTGGGAAGAGACATTCGACGATGCGTACATCAAGCTGTATGAAC‑TTTGGAAATCGAGAGGTCAATGACTATGTCAAACGTAAATACAGGTTCACTTAGTGTGGACAATAAGAAGTTTTGGGCTACCGTAGAGTCCTCGGAGc                                                                                                                                                                                                                                                                               >  1:375777/1‑301 (MQ=255)`  
                                `gagTTAGCGTACTTAGAGAAAGACCTAGCGAAAGCAGACTTCTATAAAGAACTCTTTGAGAAACATAAGGATAAATGTTATGCATAACTTCAAGTCAACCCCACCTGCCGACAGCCTATCTGATGACTTCACATCTTGCACAGAGTGGTGCCGAAAGATGTGGGAAGAGACATTCGACGATGCGTACATCAAGCTGTATGAAC‑TTTGGAAATCGAGAGGTCAATGACTATGTCAAACGTAAATACAGGTTCACTTAGTGTGGACAATAAGAAGTTTTGGGATACCGTAGAGTCCTCGGAGc                                                                                                                                                                                                                                                                               <  1:206037/301‑1 (MQ=255)`  
                                               `gagaAAGACCTAGCGAAAGCAGACTTCTATAAAGAACTCTTTGAGAAACATAAGGATAAATGTTATGCATAACTTCAAGTCAACCCCACCTGCCGACAGCCTATCTGATGACTTCACATCTTGCTCAGAGTGGTGCCGAAAGATGTGGGAAGAGACATTCGACGATGCGTACATCAAGCTGTATGAACTTTTGGAAATCGAGAGGTCAATGACTATGTCAAACGTAAATACAGGTTCACTTAGTGTGGACAATAAGAAGTTTTGGGCTACCGTAGAGTCCTCGGAGCATTCCTTCGAGGtt                                                                                                                                                                                                                                                                 >  1:463254/1‑301 (MQ=255)`  
                                                 `gaAAGACCTAGCGAAAGCAGACTTATATAAAGAACTCTTTGAGAAACATAAGGATAAATGTTATGCATAACTTCAAGTCAACCCCACCTGCCGACAGCCTATCTGATGACTTCACATCTTGCTCAGAGTGGTGCCGAAAGATGTGGGAAGAGACATTCGACGATGCGTACATCAAGCTGTATGAAC‑TTTGGAAATCGAGAGGTCAATGACTATGTCAAACGTAAATACAGGTTCACTTAGTGTGGACAATAAGAAGTTTTGGGCTACCGTAGAGTCCTCGGAGcctgtctcttatacacat                                                                                                                                                                                                                                                              >  1:274647/1‑284 (MQ=255)`  
                                                    `aGACCTAGCTAAAGCAGACTTCTATAAAGACCTCTTTGAGAAACATAAGGATAAATTTTATGCATAACTTCAAGTCAACCCCCCCTGCCGACAGCCTATCTGATGACTTCACATCTTTCTCAGAGTGGTGCCGAAAGATGTGGGAAGAGACATTCGACGATGCGTACATCAAGCTGTATGAAC‑TTTGGAAATCGAGAGGTCAATGACTATGTCAAACGTAAATACAGGTTCACTTAGTGTGGACAATAAGAAGTTTTGGGCTACCGTAGAGTCCTCGGAGCATTCCTTCGAGGTTCCAATc                                                                                                                                                                                                                                                           <  1:83770/301‑1 (MQ=255)`  
                                                    `aGACCTAGCGAAAGCATACTTCTATAAAGATCTCTTTGAGAAACATAAGGATAAATGTTATGCATAACTTCAAGTCAACCCCACCTGCCGACAGCCTATCTGATGACTTCACATCTTGCTCAGAGTGGTGACGAAAGATGTGGGAAGAGACATTCGACGATGCGTACATCAAGCTGTATGAAC‑TTTGGAAATCGAGAGGTCAATGACTATGTCAAACGTAAATACAGGTTCACTTAGTGTGGACAATAAGAAGTTTTGGGCTACCGTAGAGTCCTCGGAGCATTCCTTCGAGGTTCCAATc                                                                                                                                                                                                                                                           <  1:722567/301‑1 (MQ=255)`  
                                                     `gACCTAGCGAAAGCAGACTTCTATAAAGAACTCTTTGAGAAACATAAGGATAAATGTTATGCATAACTTCAAGTCAACCCCACCTGCCGACAGCCTATCTGATGACTTCACATCTTGCTCAGAGTGGTGCCGAAAGATGTGGGAAGAGACATTCGACGATGCGTACATCAAGCTGTATGAAC‑TTTGGAAATCGAGAGGTCAATGACTATGTCAAACGTAAATACAGGTTCACTTAGTGTGGACAATAAGAAGTTTTGGGCTACCGTAGAGTCCTCTGAGCATTCCTTCGAGGTTCCAATCt                                                                                                                                                                                                                                                          >  1:16223/1‑301 (MQ=255)`  
                                                               `aaGCAGACTTCTATAAAGAACTCTTTGAGAAACATAAGGATAAATGGTATGCATAACTTCAAGTCAACCCCACTTGCCGACAGCCCATCTGATGACTTCACATCTTGCTCAGAGTGGTGCCGAAAGATGGGGGAAGAGACATTCGACGATGCGTACATCAAGCTGTATGAAC‑CTTGGAAATCGAGAGGTCAATGACTATGTCAAACGTAAATACAGGTTCACTTAGTGTGGACAATAAGAAGTTTTGGGCTACCGTAGAGTCCTCGGAGCATTCCTTCGAGGTTCCAATCTACGCTGAGAc                                                                                                                                                                                                                                                <  1:304594/301‑1 (MQ=255)`  
                                                                `gagatgtgtataagagacagtctTTGAGAAACATAAGGATAAATGTTATGCATAACTTCAAGTCAACCCCACCTGCCGACAGCCTATCTGATGACTTCACATCTTGCTCAGAGTGGTGCCGAAAGATGTGGGAAGAGACATTCGACGATGCGTACATCAAGCTGTATGAAC‑TTTGGAAATCGAGAGGTCAATGACTATGTCAAACGTAAATACAGGTTCACTTAGTGTGGACAATAAGAAGTTTTGGGCTACCGTAGAGTCCTCGGAGCATTCCTTCGAGGTTCCAATCTACGCTGAGAcc                                                                                                                                                                                                                                               <  1:634664/281‑1 (MQ=255)`  
                                                                 `gagacagtCTATAAAGAACTCTTTGAGAAACATAAGGATAAATGTTATGCATAACTTCAAGTCAACCCCACCTGCCGACAGCCTATCTGATGACTTCACATCTTGCTCAGAGTGGTGCCGAAAGATGTTGGAAGAGACATTCGACGATGCGTACATCAAGCTGTATGAAC‑TTTGGAAATCGAGAGGTCAATGACTATGTCAAACGTAAATACAGGTTCACTTAGTGTGGACAATAAGAAGTTTTGGGCTACCGTAGAGTCCTCGGAGCATTCCTTCGAGGTTCCAATCTACGCTGAGAccc                                                                                                                                                                                                                                              <  1:736893/294‑1 (MQ=255)`  
                                                                 `gCAGACTTCTATAAAGAACTCTTTGAGAAACATAAGGATAAATGTTATGCATAACTTCAAGTCAACCCCACCTGCCGACAGCCTATCTGATGACTTCACATCTTGCTCAGAGTGGTTCCGAAAGATGTGGGAAGAGACATTCGACGATGCGTACATCAAGCTGTATGAACTTTTGGAAATCGAGAGGTCAATGACTATGTCAAACGTAAATACAGGTTCACTTAGTGTGGACAATAAGAAGTTTTGGGCTACCGTAGAGTCCTCGGAGCATTCCTTCGAGGTTCCAATCTACGCTGAGAcc                                                                                                                                                                                                                                               <  1:318589/301‑1 (MQ=255)`  
                                                                 `gCAGACTTCTATAAAGAACTCTTTGAGAAACATAAGGATAAATGTTATGCATAACTTCAAGTCAACCCCACCTGCCGACAGCCTATCTGATGACTTCACATCTTGCTCAGAGTGGTGCCGAAAGATGTGGGAAGAGACATTCGACGATGCGTACATCAAGCTGTATGAAC‑TTTGGAAATCGAGAGGTCAATGACTATGTCAAACGTAAATACAGGTTCACTTAGTGTGGACAATAAGAAGTTTTGGGCTACCGTAGAGTCCTCGGAGCATTCCTTCGAGGTTCCAATCTACGCTGAGAccc                                                                                                                                                                                                                                              <  1:125209/301‑1 (MQ=255)`  
                                                                        `tCTATAAAGAACTCTTTGAGAAACATAAGGATAAATGTTATGCATAACTTCAAGTCAACCCCACCTGCCGACAGCCTATCTGATGACTTCACATCTTGCTCAGAGTGGTGCCGAAAGATGTGGGAAGAGACATTCGACGATGCGTACATCAAGCTGTATGAACTTTTGGAAATCGAGAGGTCAATGACTATGTCAAACGTAAATACAGGTTCACTTAGTGTTGACAATAAGAAGTTTTGGGCTACCGTAGATTCCTCGGAGCATTCCTTCGAGGTTCCCATCTACGCTGATACCCtcgccc                                                                                                                                                                                                                                        >  1:455445/1‑296 (MQ=255)`  
                                                                        `tCTATAAAGAACTCTTTGAGAAACATAAGGATAAATGTTATGCATAACTTCAAGTCAACCCCACCTGCCGACAGCCTATCTGATGACTTCACATCTTGCTCAGAGTGGTGCCGAAAGATGTGGGAAGAGACATTCGACGATGCGTACATCAAGCTGTATGAAC‑TTTGGAAATCGAGAGGTCAATGACTATGTCAAACGTAAATACAGGTTCACTTAGTGTGGACAATAAGAAGTTTTGGGCTACCGTAGAGTCCTCGGAGCATTCCTTCGAGGTTCCAATCTACGCTGATACCCTAGACGa                                                                                                                                                                                                                                       >  1:486329/1‑301 (MQ=255)`  
                                                                        `tCTATAAAGAACTCTTTGAGAAACATAAGGATAAATGTTATGCATAACTTCAAGTCAACCCCACCTGCCGACAGCCTATCTGATGACTTCACATCTTGCTCAGAGTGGTGCCGAAAGATGTGGGAAGAGACATTCGACGATGCGTACATCAAGCTGTATGAAC‑TTTGGAAATCGAGAGGTCAATGACTATGTCAAACGTAAATACAGGTTCACTTAGTGTGGACAATAAGAAGTTTTGGGCTACCGTAGAGTCCTCGGAGCATTCCTTCGAGGTTCCAATCTACGCTGAGACCCTAGACGa                                                                                                                                                                                                                                       >  1:697738/1‑301 (MQ=255)`  
                                                                         `cTATAAAGAACTCTTTGAGAAACATAAGGATAAATGTTATGCATAACTTCAAGTCAACCCCACCTGCCGACAGCCTATCTGATGACTTCACATCTTGCTCAGAGTGGTGCCGAAAGATGTGGGAAGAGACATTCGACGATGCGTACATCAAGCTGTATGAAC‑TTTGGAAATCGAGAGGTCAATGACTATGTCAAACGTAAATACAGGTTCACTTAGTGTGGACAATAAGAAGTTTTGGGCTACCGTAGAGTCCTCGGAGCATTCCTTCGAGGTTCCAATcctgtctcttatacacatctcc                                                                                                                                                                                                                                      >  1:610996/1‑280 (MQ=255)`  
                                                                           `ataAAGAACTCTTTGAGAAACATAAGGATAAATGTTATGCATAACTTCAAGTCAACCCCACCTGCCGACAGCCTATCTGATGACTTCACATCTTGCTCAGAGTGGTGCCGAAAGATGTGGGAAGAGACATTCGACGATGCGTACATCAAGCTGTATGAAC‑TTTGGAAATCGAGAGGTCAATGACTATGTCAAACGTAAATACAGGTTCACTTAGTGTGGACAATAAGAAGTTTTGGGCTACCGTAGAGTCCTCGGAGCATTCCTTCGAGGTTCCAATCTACGCTGCGActgtctcttatac                                                                                                                                                                                                                                    >  1:524363/1‑289 (MQ=255)`  
                                                                           `ataAAGAACTCTTTGAGAAACATAAGGATAAATGTTATGCATAACTTCAAGTCAACCCCACCTGCCGACAGCCTATCTGATGACTTCACATCTTGCTCAGAGTGGTGCCGAAAGATGTGGGAAGAGACATTCGACGATGCGTACATCAAGCTGTATGAAC‑TTTGGAAATCGAGAGGTCAATGACTATGTCAAACGTAAATACAGGTTCACTTAGTGTGGACAATAAGAAGTTTTGGGCTACCGTAGAGTCCTCGGAGCATTCCTTCGAGGTTCCAATCTACGCTGAGACCCTAGACGAAGc                                                                                                                                                                                                                                    >  1:324857/1‑301 (MQ=255)`  
                                                                            `aaaaGAACTCTTTGAGAAACATAAGGATAAATGTTATGTATAACTTCAAGTCAACCCCACCTGCCGACAGCCTATCTGATGACTTCGCATCTTGCTCAGAGTGGTGCCGAAAGATGTGGGAAGAGACATTCGACGATGAGTACATCAAGCTGTATGAACTTTTGGAAATCGAGAGGTCAATGACTATGTCAAACGTAAATACAGGTTCACTTAGTGTGGACAATAAGAAGTTTTGGGCTACCGTAGAGTCCTCGGAGCATTCCTTCGAGGTTCCAATCTACGCTGAGACCCTAGACGAAGc                                                                                                                                                                                                                                    <  1:281826/300‑1 (MQ=255)`  
                                                                                  `aCTCTTTGAGAAACATAAGGATAAATGTTATGCATAACTTCAAGTCAACCCCACCTGCCGACAGCCTATCTGATGACTTCACATCTTGCTCAGAGTGGTGCCGAAAGATGTGGGAAGAGACATTCGACGATGCGTACATCAAGCTGTATGAAC‑TTTGGAAATCGAGAGGTCAATGACTATGTCAAACGTAAATACAGGTTCACTTAGTGTGGACAATAAGAAGTTTTGGGCTACCGTAGAGTCCTCGGAGCATTCCTTCGAGGTTCCAATCTACGCTGAGACCCTAGACGAAGCTCTGGAg                                                                                                                                                                                                                             <  1:259729/301‑1 (MQ=255)`  
                                                                                  `aCTCTTTGAGAAACATAAGGATAAATGTTATGCATAACTTCAAGTCAACCCCACCTGCCGACAGCCTATCTGATGACTTCACATCTTGCTCAGAGTGGTGCCGAAAGATGTGGGAAGAGACATTCGACGATGCGTACATCAAGCTGTATGAAC‑TTTGGAAATCGAGAGGTCAATGACTATGTCAAACGTAAATACAGGTTCACTTAGTGTGGACAATAAGAAGTTTTGGGCTACCGTAGAGTCCTCGGAGCATTCCTTCGAGGTTCCAATCTACGCTGAGACCCTAGACGAAGCTCTGGAg                                                                                                                                                                                                                             <  1:471098/301‑1 (MQ=255)`  
                                                                                       `ttGAGAAACATAAGTATAAATGTTATGCATAACTTCAAGTCAACCCCACCTGCCGACAGCCTATCTGATGACTTCACATCTTGCTCAGAGTGGTGCCGAAAGATGTGGGAAGAGACATTCGACGATGCGTACATCAAGCTGTATGAAC‑TTTGGAAATCGAGAGGTCAATGACTATGTCAAACGTAAATACAGGTTCACTTAGTGTGGACAATAAGAAGTTTTGGGCTACCGTAGAGTCCTCGGAGCATTCCTTCGAGGTTCCAATCTACGCTGAGACCCTAGACGAAGCTCTGGAGTTAGc                                                                                                                                                                                                                        <  1:580349/301‑1 (MQ=255)`  
                                                                                       `ttGAGAAACATAAGGATAAATGTTATGCATAACTTCAAGTCAACCCCACCTGCCGACAGCCTATCTGATGACTTCACATCTTGCTCAGAGTGGTGCCGAAAGATGTGGGAAGAGACATTCGACGATGCGTACATCAAGCTGTATGAAC‑TTTGGAAATCGAGAGGTCAATGACTATGTCAAACGTAAATACAGGTTCACTTAGTGTGGACAATAAGAAGTTTTGGGCTACCGTAGAGTCCTCGGAGCATTCCTTCGAGGTTCCAATCTACGCTGAGACACTAGACGAAGCTCTGGAGTTAGc                                                                                                                                                                                                                        <  1:822486/301‑1 (MQ=255)`  
                                                                                        `tgggaAACATAAGGATAAATGTTATGCATAACTTCAAGTCAACCCCCCCTGCCGACAGCCTATCTGATGACTTCACATCTTGCTCAGAGTGGTGCCGAAAGATGTGGGAAGAGACATTCGACGATTCGTACATCAAGCTGTATGAAC‑TTTGGAAATCGAGAGGTCAATGACTATGTCAAACGTAAATACAGGTTCACTTAGTGTGGACAATAAGAAGTTTTGGGCTACCGTAGAGTCCTCGGAGCATTCCTTCGAGGTTCCAATCTACGCTGAGACCCTAGACGAAGCTCTGGAGTTAGcc                                                                                                                                                                                                                       <  1:367414/298‑1 (MQ=255)`  
                                                                                              `aCATAAGGATAAATGTTATGCATAACTTCAAGTCAACCCCACCTGCCGACAGCCTATCTGATGACTTCACATCTTGCTCAGAGTGGTGCCGAAAGATGTGGGAAGAGACATTCGACGATGCGTACATCAAGCTGTATGAAC‑TTTGGAAATCGAGAGGTCAATGACTATGTCAAACGTAAATACAGGTTCACTTAGTGTGGACAATAAGAAGTTTTGGGCTACCGTAGAGTCCTCGGAGCATTCCTTCGAGGTTCCAATCTACGCTGAGACCCTAGACTAAGCTCTGGAGTTAGCCGAATgg                                                                                                                                                                                                                 >  1:555841/1‑301 (MQ=255)`  
                                                                                              `aCATAAGGATAAATGTTATGCATAACTTCAAGTCAACCCCACCTGCCGACAGCCTATCTGATGACTTCACATCTTGCTCAGAGTGGTGCCGAAAGATGTGGGAAGAGACATTCGACGATGCGTACATCAAGCTGTATGAAC‑TTTGGAAATCGAGAGGTCAATGACTATGTCAAACGTAAATACAGGTTCACTTAGTGTGGACAATAAGAAGTTTTGGGCTACCGTAGAGTCCTCGGAGCATTCCTTCGAGGTTCCAATCTACGCTGAGACCCTAGACGAAGCTCTGGAGTTAGCCGAATgg                                                                                                                                                                                                                 <  1:228325/301‑1 (MQ=255)`  
                                                                                                 `aaaGGATAAATGTTATGCAGAACTTCAAGTCAACCCCACCTGCCGACAGCCTATCTGATGACTTCACATCTTGCTCAGAGTGGTGACGAAAGATGTGGGAAGAGACATTCGACGATGCGTACATCAAGCTGTATGAAC‑TTTGGAAATCGAGAGGTCAATGACTATGTCAAACGTAAATACAGGTTCACTTAGTGTGGACAATAAGAAGTTTTGGGCTACCGTAGAGTCCTCGGAGCATTCCTTCGAGGTTCCAATCTACGCTGAGACCCTAGACGAAGCTCTGGAGTTAGCCGAATGGCaa                                                                                                                                                                                                              <  1:197607/300‑1 (MQ=255)`  
                                                                                                    `ggATAAATGTTATGCATAACTTCAAGTCAACCCCACCTGCCGACAGCCTATCTGATGACTTCACATCTTGCTCAGAGTGGTGCCGAAAGATGTGGGAAGAGACATTCGACGATGCGTACATCAAGCTGTATGAAC‑TTTGGAAATCGAGAGGTCAATGACTATGTCAAACGTAAATACAGGTTCACTTAGTGTGGACAATAAGAAGTTTTGGGCTACCGTAGAGTCCTCGGAGCATTCCTTCGAGGTTCCAATCTACGCTGAGACCCTAGACGAAGCTCTGGAGTTAGcctgtctcttatac                                                                                                                                                                                                           >  1:108257/1‑289 (MQ=255)`  
                                                                                                     `gATAAATGTTATGCATAACTTCAAGTCAACCCCACCTGCCGACAGCCTATCTGATGACTTCACATCTTGCTCAGAGTGGTGCCGAAAGATGTGGGAAGAGACATTCGACGATGCGTACATCAAGCTGTATGAAC‑TTTGGAAATCGAGAGGTCAATGACTATGTCAAACGTAAATACAGGTTCACTTAGTGTGGACAATAAGAAGTTTTGGGCTACCGTAGAGTCCTCGGAGCATTCCTTCGAGGTTCCAATCTACGCTGAGACCCTAGACGAAGCTCTGGAGTTAGCCGAATGGCAATACg                                                                                                                                                                                                          >  1:707960/1‑301 (MQ=255)`  
                                                                                                     `aaTAAATGTTATGCATAACTTCAAGTCAACCCCACCTGCCGACAGCCTATCTGATGACTTCACATCTTGCTCAGGGTGGTGCCGAAAGATGTGGGAAGAGACATTCGACGATGCGTACATCAAGCTGTATGAAC‑TTTGGAAATCGAGAGGTCAATGACTATGTCAAACGTAAATACAGGTTAACTTAGTGTGGACAATAAGAAGTTTTGGGCTACCGTAGAGTCCTCGGAGCATTCCTTCGAGGTTCCAATCTACGCTGAGACCCTAGACGAAGCTCTGGAGTTAGCCGAATGGCAATACg                                                                                                                                                                                                          <  1:411078/300‑1 (MQ=255)`  
                                                                                                          `aTGTTATGCATAACTTCAAGTCAACCCCACCTGCCGACAGCCTATCTGATGACTTCACATCTTGCTCAGAGTGGTGCCGAAAGATGTGGGAAGAGACATTCGACGATGCGTACATCAAGCTGTATGAAC‑TTTGGAAATCGAGAGGTCAATGACTATGTCAAACGTAAATACAGGTTCACTTAGTGTGGACAATAAGAAGTTTTGGGCTACCGTAGAGTCCTCGGAGCATTCCTTCGAGGTTCCAATCTACGCTGAGACCCTAGACGAAGCTCTGGAGTTAGCCGAATGGCAATACGTTCCg                                                                                                                                                                                                     >  1:91075/1‑301 (MQ=255)`  
                                                                                                            `tttATGCATAACTTCAAGTCACCCCCACCTCCCTACAGCCTATCTGATGACTTCACATCTTGCTCAGAGTGGTGCCGAAAGATGTGGGAAGAGACATTCGACGATGCGTACATCAAGCTGTATGAAC‑TTTGGAAATCGAGAGGTCAATGACTATGTCAAACGTAAATACAGGTTCACTTAGTGTGGACAATAAGAAGTTTTGGGCTACCGTAGAGTCCTCGGAGCATTCCTTCGAGGTTCCAATCTACGCTGAGACCCTAGACGAAGCTCTGGAGTTAGCCGAATGGCAATACGTTCCggc                                                                                                                                                                                                   <  1:808461/300‑1 (MQ=255)`  
                                                                                                                      `aCTTCAAGTCAACCCCACCTGCCGACAGCCTATCTGATGACTTCACATCTTGCTCAGAGTGGTTCCGAAAGATGTGGGAAGAGACATTCGACGATGCGTACATCAAGCTGTATGAAC‑TTTGGAAATCGAGAGGTCAATGACTATGTCAAACGTAAATACAGGTTCACTTAGTGTGGACAATAAGAAGTTTTGGGCTACCGTAGAGTCCTCGGAGCATTCCTTCGAGGTTCCAATCTACGCTGAGACCCTAGACGAAGCTCTGGAGTTAGCCGAATGGCAATACGTTCCGGCTGGCTTTGAg                                                                                                                                                                                         >  1:539645/1‑301 (MQ=255)`  
                                                                                                                       `cTTCAAGTCAACCCCACCTGCCGACAGCCTATTTGATGACTTCACATCTTGCTCAGAGTGGTGCCGAAAGATGTGGGAAGAGACATTCGACGATGCGTACATCAAGCTGTATGAAC‑TTTGGAAATCGAGAGGTCAATGACTATGTCAAACGTAAATACAGGTTCACTTAGTGTGGACAATAAGAAGTTTTGGGCTACCGTAGAGTCCTCGGAGCATTCCTTCGAGGTTCCAATCTACGCTGAGACCCTAGACGAAGCTCTGGAGTTAGCCGAATGGCAATACGTTCCGGCTGGCTTTGAgg                                                                                                                                                                                        >  1:626886/1‑301 (MQ=255)`  
                                                                                                                       `cTTCAAGTCAACCCAACCTGCCGACAGCCTATCTGATGACTTCACATCTTGCTCAGAGTGGTGCCGAAAGATGTGGGAAGAGACATTCGACGATGCGTACATCAAGCTGTATGAAC‑TTTGGAAATCGAGAGGTCAATGACTATGTCAAACGTAAATACAGGTTCACTTAGTGTGGACAATAAGAAGTTTTGGGCTACCGTAGAGTCCTCGGAGCATTCCTTCGAGGTTCCAATCTACGCTGAGACCCTAGACGAAGCTCTGGAGTTAGCCGAATGGCAATACGTTCCGGCTGGCTTTGAgg                                                                                                                                                                                        <  1:662180/301‑1 (MQ=255)`  
                                                                                                                       `agacaggTCAACCCCACCTGCCGACAGCCTATCTGATGACTTCACATCTTGCTCAGAGTGGTGCCGAAAGATGTGGGAAGAGACATTCGACGATGCGTACATCAAGCTGTATGAAC‑TTTGGAAATCGAGAGGTCAATGACTATGTCAAACGTAAATACAGGTTCACTTAGTGTGGACAATAAGAAGTTTTGGGCTACCGTAGAGTCCTCGGAGCATTCCTTCGAGGTTCCAATCTACGCTGAGACCCTAGACGAAGCTCTGGAGTTAGCCGAATGGCAATACGTTCCGGATGGCTTTGAgg                                                                                                                                                                                        <  1:715651/295‑1 (MQ=255)`  
                                                                                                                                     `cACCTGCCGACAGCCTATCTGATGACTTCACATCTTGCTCAGAGTGGTGCCGAAAGATGTGGGAAGAGACATTCGACGATGCGTACATCAAGCTGTATGAAC‑TTTGGAAATCGAGAGGTCAATGACTATGTCAAACGTAAATACAGGTTCACTTAGTGTGGACAATAAGAAGTTTTGGGCTACCGTAGAGTCCTCGGAGCATTCCTTCGAGGTTCCAATCTACGCTGAGACCCTAGACGAAGCTCTGGAGTTAGCCGAATGGCAATACGTTCCGGCTGGCTTTGAGGTTACTCGTGTGCGt                                                                                                                                                                          >  1:191001/1‑301 (MQ=255)`  
                                                                                                                                               `cAGCCTATCTGATGACTTCACATCTTGCTCAGAGTGGTGCCGAAAGATGTGGGAAGAGACATTCGACGATGCGTACATCAAGCTGTATGAAC‑TTTGGAAATCGAGAGGTCAATGACTATGTCAAACGTAAATACAGGTTCACTTAGTGTGGACAATAAGAAGTTTTGGGCTACCGTAGAGTCCTCGGAGCATTCCTTCGAGGTTCCAATCTACGCTGAGACCCTAGACGAAGCTCTGGAGTTAGCCGAATGGCAATACGTTCCGGCTGGCTTTGAGGTTACTCGTGTGCGTCCTTGTGTAg                                                                                                                                                                <  1:146542/301‑1 (MQ=255)`  
                                                                                                                                                  `ccTATCTGATGACTTCACATCTTGCTCAGAGTGGTGCCGAAAGATGTGGGAAGAGACATTCGACGATGCGTACATCAAGCTGTATTAAC‑TTTGGAAATCGAGAGGTCAATGACTATGTCAAACGTAAATACAGGTTCACTTAGTGTGGACAATAAGAAGTTTTGGGCTACCGTAGAGTCCTCGGAGCATTCCTTCGAGGTTCCAATCTACGCTGAGACCCTAGACGAATCTCTGGAGTTAGCCGAATGGCAATACGTTCCGGCTGGCTTTGAGGTTACTCGTGTGCGTCCTTGTGTAGCAc                                                                                                                                                             >  1:602733/1‑301 (MQ=255)`  
                                                                                                                                                  `ccTATCTGATGACTTCACATCTTGCTCAGAGTGGTGCCGAAAGATGTGGGAAGAGACATTCGACGATGCGTACATCAAGCTGTATGAAC‑TTTGGAAATCGAGAGGTCAATGACTATGTCAAACGTAAATACAGGTTCACTTAGTGTGGACAATAAGAAGTTTTGGGCTACCGTAGAGTCCTCGGAGCATTCCTTCGAGGTTCCAATCGACGCTGAGACCCTAGACGAAGCTCTGGAGTTAGCCGAATGGCAATACGTTCCGGCTGGCTTTGAGGTTACTCGTGTGCGTCCTTGTGTAGCAc                                                                                                                                                             <  1:311219/301‑1 (MQ=255)`  
                                                                                                                                                     `aTCTGATGACTTCACATCTTGCTCAGAGTGGTGCCGAAAGATGTGGGAAGAGACATTCGACGATGCGTACATCAAGCTGTATGAAC‑TTTGGAAATCGAGAGGTCAATGACTATGTCAAACGTAAATACAGGTTCACTTAGTGTGGACAATAAGAAGTTTTGGGCTACCGTAGAGTCCTCGGAGCATTCCTTCGAGGTTCCAATCTACGCTGAGACCCTAGACGAAGCTCTGGAGTTAGCCGAATGGCAATACGTTCCGGCTGGCTTTGAGGTTACTCGTGTGCGTCCTTGTGTAGCACCGa                                                                                                                                                          >  1:252961/1‑301 (MQ=255)`  
                                                                                                                                                        `tgatgaCTTCACATCTTGCTCAGAGTGGTGCCGAAAGATGTGGGAAGAGACATTCGACGATGCGTACATCAAGCTGTATGAAC‑TTTGGAAATCGAGAGGTCAATGACTATGTCAAACGTAAATACAGGTTCACTTAGTGTGGACAATAAGAAGTTTTGGGCTACCGTAGAGTCCTCGGAGCATTCCTTCGAGGTTCCAATCTACGCTGAGACCCTAGACGAAGCTCTGGAGTTAGCCGAATGGCAATACGTTCCGGCTGGCTTTGAGGTTACTCGTGTGCGTCCTTGTGTAGCACCAAAGt                                                                                                                                                       <  1:153858/301‑1 (MQ=255)`  
                                                                                                                                                             `aCTTCACATCTTGCTCAGAGTGGTGCCGAAAGATGTGGGAAGAGACATTCGACGATGCGTACATCAAGCTGTATGAAC‑TTTGGAAATCGAGAGGTCAATGACTATGTCAAACGTAAATACAGGTTCACTTAGTGTGGACAATAAGAAGTTTTGGGCTACCGTAGAGTCCTCGGAGCATTCCTTCGAGGTTCCAATCTACGCTGAGACCCTAGACGAAGCTCTGGAGTTAGCCGAATGGCAATACGTTCCGGCTGGCTTTGAGGTTACTCGTGTGCGTCCTTGTGTAGCACCGAAGTAATAc                                                                                                                                                  <  1:494368/301‑1 (MQ=255)`  
                                                                                                                                                             `aCTTCACATCTTGCTCAGAGTGGTGCCGAAAGATGTGGGAAGAGACATTCGACGATGCGTACATCAAGCTGTATGAAC‑TTTGGAAATCGAGAGGTCAATGACTATGTCAAACGTAAATACAGGTTCACTTAGTGTGGACAATAAGAAGTTTTGGGCTACCGTAGAGTCCTCGGAGCATTCCTTCGAGGTTCCAATCTACGCTGAGACCCTAGACGAAGCTCTGGAGTTAGCCGAATGGCAATACGTTCCGGCTGGCTTTGAGGTTACTCGTGTGCGTCCTTGTGTAGCACCGAAGTAATAc                                                                                                                                                  >  1:425673/1‑301 (MQ=255)`  
                                                                                                                                                             `aCTTCACAGCTTGCTCAGAGTGGTGCCGAAAGATGTGGGAAGAGACATTCGACGATGCGTACATCAAGCTGTATGAAC‑TTTGGAAATCGAGAGGTCAATGACTATGTCAAACGTAAATACAGGTTCACTTAGTGTGGACAATAAGAAGTTTTGGGATACCGTAGAGTCCTCGGAGCATTCCTTCGAGGTTCCAATCTACGCTGAGACCCTAGACGAAGCTCTGGAGTTAGCCGAATGGCAATACGTTCCGGCTGGCTTTGAGGTTACTCGTGTGCGTCCTTGTGTAGCACCGAAGTAATAc                                                                                                                                                  <  1:745397/301‑1 (MQ=255)`  
                                                                                                                                                              `cTTCACATCTTGCTCAGAGTGGTGCAGAAAGATGTGGGAAGAGACATTCGACGATGCGTACATCAAGCTGTATGAACTTTTGGAAATCGAGAGGTCAATGACTATGTCAAACGTAAATACAGGTTCACTTAGTGTGGACAATAAGAAGTTTTGGGCTACCGTAGAGTCCTCGGAGCATTCCTTCGAGGTTCCAATCTACGCTGGGACCCTAGACGAAGCTCTGGATTTAGCCGAATGGCATTACGTTCCGGCTGGCTTTGAGGTTACTCGTGTGCGTCCTTGTTTATCACCGAAGTAATAc                                                                                                                                                  >  1:440620/1‑301 (MQ=255)`  
                                                                                                                                                                    `aTCTTGCTCAGAGTGGTGCCGAAAGATGTGGGAAGAGACATTCGACGATGTGTACATCAAGCTGTATGAAC‑TTTGGAAATCGAGAGGTCAATGACTATGTCAAACGTAAATACAGGTTCGCTTAGTGTGGACAATAAGAAGTTTTGGGCTACCGTAGAGTCCTCGGAGCATTCCTTCGAGGTTCCAATCTACGCTGAGACCCTAGACGAAGCTCTGGAGTTAGCCGAATGGCAATACGTTCCGGCTGGCTTTGAGGTTACTCGTGTGCGTCCTTGTGTAGCACCGAAGTAATAcctgtctc                                                                                                                                           >  1:531835/1‑294 (MQ=255)`  
                                                                                                                                                                    `aTCTTGCTCAGAGTGGTGCCGAAAGATGTGGGAAGAGACATTCGACGATGCGTACATCAAGCTGTATGAAC‑TTTGGAAATCGAGAGGTCAATGACTATGTCAAACGTAAATACAGGTTCACTTAGTGTGGACAATAAGAAGTTTTGGGCTACCGTAGAGTCCTCGGAGCATTCCTTCGAGGTTCCAATCTACGCTGAGACCCTAGACGAAGCTCTGGAGTTAGCCGAATGGCAATACGTTCCGGCTGGCTTTGAGGTTACTCGTGTGCGTCCTTGTGTAGCACCGAAGTAATCCGACTCAc                                                                                                                                           >  1:64689/1‑301 (MQ=255)`  
                                                                                                                                                                        `tGCTCAGAGTGGTGCCGAAAGATGTGGGAAGAGACATTCGACGATGCGTACATCAAGCTGTATGAACTTTTGGAAATCGAGAGGTCAATGACTATGTCAAACGTAAATACAGGTTCACTTAGTGTGGACAATAAGAAATTTTGGGCTACCGTAGAGTCCTCGGAGCATTCCTTCGAGGTTCCAATCTACGCTGAGACCCTAGACGAAGCTCTGGAGTTAGCCGAATGGCAATACGTTCCGGCTGGCTTTGAGGTTACTCGTGTGCGTCCTTGTGTAGCACCGAAGTAATACGACTCACTAt                                                                                                                                        >  1:330914/1‑301 (MQ=255)`  
                                                                                                                                                                         `gCTCAGAGTGGTGCCGAAAGATGTGGGAAGAGACATTCGACGATGCGTACATCAATCTGTATGAAC‑TTTGGAAATCGAGAGGTCAATGACTATGTCAAACGTAAATACAGGTTCACTTAGTGTGGACAATAAGAAGTTTTGGGCTACCGTAGAGTCCTCGGAGCATTCCTTCGAGGTTCCAATCTACGCTGAGACCCTAGACGAAGCTCTGGAGTTAGCCGAATGGCAATACGTTCCGGCTGGCTTTGAGGTTACTCGTGTGCGTCCTTGTGTAGCACCGAAGTAATACGACTCACTAttt                                                                                                                                      >  1:595533/1‑300 (MQ=255)`  
                                                                                                                                                                         `gCTCAGAGTGGTGCCGAAAGATGTGGGAAGAGACATTCGACGATGCGTACATCAAGCTGTATGAAC‑TTTGGAAATCGAGAGGTCAATGACTATGTCAAACGTAAATACAGGTTCACTTAGTGTGGACAATAAGAAGTTTTGGGCCACCGTAGAGTCCTCGGAGCATTCCTTCGAGGTTCCAATCTACGCTGAGACCCTAGACGAAGCTCTGGAGTTAGCCGAATGGCAATACGTTCCGGCTGGCTTTGAGGTTACTCGTGTGCGTCCTTGTGTAGCACCGAAGTAATACGACTCACTATTa                                                                                                                                      >  1:353192/1‑301 (MQ=255)`  
                                                                                                                                                                            `agatgtgtataagagacaggtgGGAAGAGACCTTCGACGATGCGTACATCAAGCTGTATGAAC‑TTTGGAAATCGAGAGGTCAATGACTATGTCAAACGTAAATACAGGTTCACTTAGTGTGGACAATAAGAAGTTTTGGGCTACCGTAGAGTCCTCGGAGCATTCCTTCGAGGTTCCAATCTACGCTGAGACCCTAGACGAAGCTATGGAGTTAGCCGAATGGCAATACGTTCCGGCTGGCTTTGAGGTTACTCGTGTGCGTCCTTGTGTAGCACCGAAGTAATACGACTCACTATTAggg                                                                                                                                   <  1:668074/282‑1 (MQ=255)`  
                                                                                                                                                                             `ggagTGGTGCCGAAAGATGTGGGAAGAGACATTCGACGATGCGTACATCAAGCTGTATGAAC‑TTTGGAAATCGAGAGGTCAATGACTATGTCAAACGTAAATACAGGTTCACTTAGTGTGGACAATAAGAAGTTTTGGGCTACCGTAGAGTCCTCGGAGCATTCCTTCGAGGTTCCAATCTACGCTGAGACCCTAGACGAAGCTCTGGAGTTAGCCGAATGGCAATACGTTCCGGCTGGCTTTGAGGTTACTCGTGTGCGTCCTTGTGTAGCACCGAAGTAATACGACTCACTATTAGGGa                                                                                                                                  <  1:768300/300‑1 (MQ=255)`  
                                                                                                                                                                              `gagTGGTGCCGAAAGATGTGGGAAGAGACATTCGACGATGCGTACATCAAGCTGTATGAAC‑TTTGGAAATCGAGAGGTCAATGACTATGTCAAACGTAAATACAGGTTCACTTAGTGTGGACAATAAGAAGTTTTGGGCTACCGTAGAGTCCTCGGAGCATTCCTTCGAGGTTCCAATCTACGCTGAGACCCTAGACGAAGCTCTGGAGTTAGCCGAATGGCAATACGTTCCGGCTGGCTTTGAGGTTACTCGTGTGCGTCCTTGTGTAGCACCGAAGTAATACGACTCACTATTAGGGaa                                                                                                                                 >  1:641578/1‑301 (MQ=255)`  
                                                                                                                                                                              `gagTGGTGCCGAAAGATGTGGGAAGAGACATTCGACGATGCGTACATCAAGCTGTATGAAC‑TTTGGAAATAGAGAGGTCAATGACTATGTCAAACGTAAATACAGGTTCACTTAGTGTGGACAATAAGAAGTTTTGGGCTACCGTAGAGTCCTCGGAGCATTCCTTCGAGGTTCCAATCTACGCTGAGACCCTAGACGAAGCTCTGGAGTTAGCCGAATGGCAATACGTTCCGGCTGGCTTTGAGGTTACTCGTGTGCGTCCTTGTGTAGCACCGAAGTAATACGACTCACTATTAGGGaa                                                                                                                                 <  1:177293/301‑1 (MQ=255)`  
                                                                                                                                                                               `agacaggCCGAAAGATGTGGGAAGAGACATTCGACGATGCGTACATCAAGCTGTATGAAC‑TTTGGAAATCGAGAGGTCAATGACTATGTCAAACGTAAATACAGGTTCACTTAGTGTGGACAATAAGAAGTTTTGGGCTACCGTAGAGTCCTCGGAGCATTCCTTCGAGGTTCCAATCTACGCTGAGACCCTAGACGAAGCTCTGGAGTTAGCCGAATGGCAATACGTTCCGGCTGGCTTTGAGGTTACTCGTGTGCGTCCTTGTGTAGCACCGAAGTAATACGACTCACTATTAGGGAAg                                                                                                                                <  1:100427/295‑1 (MQ=255)`  
                                                                                                                                                                                 `tggtgCCGAAAGATGTGGGAAGAGACATTCGACGATGCGTACATCAAGCTGTATGAAC‑TTTAGAAATCGAGAGGTCAATGACTATGTCAAACGTAAATACAGGTTCACTTAGTGTGGACAATAAGAAGTTTTGGGCTACCGTAGAGTCCTCGGAGCATTCCTTCGAGGTTCCAATCTACGCTGAGACCCTAGACGAAGCTCTGGAGTTAGCCGAATGGCAATACGTTCCGGCTGGCTTTGAGGTTACTCGTGTGCGTCCTTGTGTAGCACCGAAGTAATACGACTCACTATTAGGGAAGAc                                                                                                                              <  1:509271/301‑1 (MQ=255)`  
                                                                                                                                                                                            `gATGTGGGAAGAGACATTCGACGATGCGTACATCACGATGTATGAAC‑TTTGGAAATCGAGAGGTCAATGACTATGTCAAACGTAAATACAGGTTCACTTAGTGTGGACAATAAGAAGTTTTGGGCTACCGTAGAGTCCTCGGAGCATTCCTTCGAGGTTCCACTCTACGCTGAGACCCTCGACGAAGCTCTGGCGTTATCCGAATGGCAACACGTTCCGGCTGGCGTTGAGGTTACTCGTGTGCGTCCTTGTGTAGCACCGAAGTCATACGCCTCACTATTAGGGAAGACTCCATCTgaga                                                                                                                   >  1:802672/1‑301 (MQ=255)`  
                                                                                                                                                                                            `gATGTGGGAAGAGACATTCGACGATGCGTACATCAAGCTGTATGAAC‑TTTGGAAATCGAGAGGTCAATGACTATGTCAAACGTAAATACAGGTTCACTTAGTGTGGACAATAAGAAGTTTTGGGCTACCGTAGAGTCCTCGGAGCATTCCTTCGAGGTTCCAATCTACGCTGAGACCCTAGACGAAGCTCTGGAGTTAGCCGAATGGCAATACGTTCCGGCTGGCTTTGAGGTTACTCGTGTGCGTCCTTGTGTAGCACCGAAGTAATACGACTCACTATTAgggctgtctcttatacaca                                                                                                                   >  1:619320/1‑285 (MQ=255)`  
                                                                                                                                                                                               `gtgGGAAGAGACATTCGACGATGCGTACATCAAGCTGTATGAAC‑TTTGGAAATCGAGAGGTCAATGACTATGTCAAACGTAAATACAGGTTCACTTAGTGTGGACAATAAGAAGTTTTGGGCTACCGTAGAGTCCTCGGAGCATTCCTTCGAGGTTCCAATCTACGCTGAGACCCTAGACGAAGCTCTGGAGTTAGCCGAATGGCAATACGTTCCGGCTGGCTTTGAGGTTACTCGTGTGCGTCCTTGTGTAGCACCGAAGTAATACGACTCACTATTAGGGAAGACTCCCTCTGAGAAAc                                                                                                                <  1:295946/301‑1 (MQ=255)`  
                                                                                                                                                                                                                `acgaTGCGTACATCAAGCTGTATGAAC‑TTTGGAAATCGAGAGGTCAATGACTATGTCAAACGTAAATACAGGTTCACTTAGTGTGGACAATAAGAAGTTTTGGGCTACCGTAGAGTCCTCGGAGCATTCCTTCTAGGTTCCAATCTACGCTGAGACCCTAGACGAAGCTCTGGAGTTAGCCGAATGGCAATACGTTCCGGCTGGCTTTGAGGTTACTCGTGTGCGTCCTTGTGTAGCACCGAAGTAATACGACTCACTATTAGGGAAGACTCCCTCTGAGAAACCAAACGAAACCTAAAgg                                                                                               <  1:447048/301‑1 (MQ=255)`  
                                                                                                                                                                                                                    `tGCGTACATCAAGCTGTATGAACTTTTGGAAATCGAGAGGTCAATGACTATGTCAAACGTAAATACAGGTTCACTTAGTGTGGACAATAAGAAGTTTTGGGCTACCGTAGAGTCCTCGGAGCATTCCTTCGAGGTTCCAATCTACGCTGAGACCCTAGACGAAGCTCTGGAGTTAGCCGAATGGCAATACGTTCCGGCTGGCTTTGAGGTTACTCGTGTCCGTCCTTGTGTAGCATCGAAGTAATACGACTCACTATTAGGGAAGACTCCCTCTGAGAAACCAAACGAAACCTAAAGgaga                                                                                            >  1:775280/1‑301 (MQ=255)`  
                                                                                                                                                                                                                    `tGCGTACATCAAGCTGTATGAAC‑TTTGGAAATCGAGAGGTCAATGACTATGTCAAACGTAAATACAGGTTCACTTAGTGTGGACAATAAGAAGTTTTGGGCTACCGTAGAGTCCTCGGAGCATTCCTTCGAGGTTCCAATCTACGCTGAGACCCTAGACGAAGCTCTGGAGTTAGCCGAATGGCAATACGTTCCGGCTGGCTTTGAGGTTACTCGTGTGCGTCCTTGTGTAGCACCGAAGTAATACGACTCACTATTAGGGAAGACTCCCTCTGAGAAACCAAACGAAACCTAAAggctgt                                                                                           >  1:212986/1‑297 (MQ=255)`  
                                                                                                                                                                                                                     `gCGTACATCAAGCTGTATGAAC‑TTTGGAAATCGAGAGGTCAATGACTATGTCAAACGTAAATACAGGTTCACTTAGTGTGGACAATAAGAAGTTTTGGGCTACCGTAGAGTCCTCGGAGCATTCCTTCGAGGTTCCAATCTACGCTGAGACCCTAGACGAAGCTCTGGAGTTAGCCGAATGGCAATACGTTCCGGCTGGCTTTGAGGTTACTCGTGTGCGTCCTTGTGTAGCACCGAAGTAATACGACTCACTATTAGGGAAGACTCCCTCTGAGAAACCAAACGAAACCTAAAGTAGAtt                                                                                          >  1:418303/1‑301 (MQ=255)`  
                                                                                                                                                                                                                                 `cTGTATGAAC‑TTTGGAAATCGAGAGGTCAATGACTATGTCAAACGTAAATACAGGTTCACTTAGTGTGGACAATAAGAAGTTTTGGGCTACCGTAGAGTCCTCGGAGCATTCCTTCGAGGTTCCAATCTACGCTGAGACCCTAGACGAAGCTCTGGAGTTAGCCGAATGGCAATACGTTCCGGCTGGCTTTGAGGTTACTCGTGTGCGTCCTTGTGTAGCACCGAAGTAATACGACTCACTATTAGGGAAGACTCCCTCTGAGAAACCAAACGAAACCTAAAGGAGATTAACATTATGGCt                                                                              >  1:237533/1‑301 (MQ=255)`  
                                                                                                                                                                                                                                   `gTATGAAC‑TTTGGAAATCGAGAGGTCAATGACTATGTCATACGTAAATACAGGTTCACTTAGTGTGGACAATAAGAAGTTTTGGGCTACCGTAGAGTCCTCGGAGCATTCCTTCGAGGTTCCAATCTACGCTGAGACCCTAGACGAAGCTCTGGAGTTAGCCGAATGGCAATACGTTCCGGCTGGCTTTGAGGTTACTCGTGTGCGTCCTTGTGTAGCACCGAAGTAATACGACTCACTATTAGGGAAGACTCCCTCTGAGAAACCAAACGAAACCTAAAGGAGATTAACATTATGGCTaa                                                                            >  1:3962/1‑301 (MQ=255)`  
                                                                                                                                                                                                                                   `gTATGAAC‑TTTGGAAATCGAGAGGTCAATGACTATGTCAAACGTAAATACAGGTTCACTTAGTGTGGACAATAAGAAGTTTTGGGCTACCGTAGAGTCCTCGGAGCATTCCTTCGAGGTTCCAATCTACGCTGAGACCCTAGACGAAGCTCTGGAGTTAGCCGAATGGTAATACGTTCCGGCTGGCTTTGAGGTTACTCGTGTGCGTCCTTGTGTAGCACCGAAGTAATACGACTCACTATTAGGGAAGACTCCCTCTGAGAAACCAAACGAAACCTAAAGGAGATTAACATTATGGCTaa                                                                            >  1:220877/1‑301 (MQ=255)`  
                                                                                                                                                                                                                                   `gTATGAAC‑GTTGGAAATCGAGAGGTCAATGACTATGTCAAACGTAAATACAGGTTCACTTAGTGTGGACAATAAGAAGTTTTGGGCTACCGTAGAGTCCTCGGAGCATTCCTTCGAGGTTCCAATCTACGCTGAGACCCTAGACGAAGCTCTGGAGTTAGCCGAATGGCAATACGTTCCGGCTGGCTTTGAGGTTACTCGTGTGCGTCCTTGTGTAGCACCGAAGTAATACGACTCACTATTAGGGACGACTCCCTCTGAGAAACAAACCGAAACCTAAAGGAGATTAAccagtctcttaa                                                                            >  1:564125/1‑290 (MQ=255)`  
                                                                                                                                                                                                                                   `gTATGAAC‑CTTGGAAATCGAGAGGTCAATGACTATGTCAAACGTAAATACAGGTTCACTTAGTGTGGACAATAAGAAGTTTTGGGCTACCGTAGAGTCCTCGGAGCATTCCTTCGAGGTTCCAATCTACGCTGAGACCCTAGACGAAGCTCTGGAGTTAGCCGAATGGCAATACGTTCCGGCTGGCTTTGAGGTTACTCGTGTGCGTCCTTGTGTAGCACCGAAGTAATACGACTCACTATTAGGGAAGACTCCCTCTGAGAAACCAAACGAAACCTAAAGGAGATTAACATTATGGcctg                                                                            >  1:683842/1‑298 (MQ=255)`  
                                                                                                                                                                                                                                                `gAAATCGAGAGGTCAATGACTATGTCAAACGTAAATACAGGTTCACTTAGTGTGGACAATAAGAAGTTTTGGGCTACCGTAGAGTACTCGGAGCATTCCTTCGAGGTTCCAATCTACGCTGAGACCCTAGACGAAGCTCTGGAGTTAGCCGAATGGCAATACGTTCCGGCTGGCTTTGAGGTTACTCGTGTGCGTCCTTGTGTAGCACCGAAGTAATACGACTCACTATTAGGGAAGACTCCCTCTGAGAAACCAAACGAAACCTAAAGGAGATTAACATTATGGCTAAGAAGATTTTCAc                                                                <  1:403703/301‑1 (MQ=255)`  
                                                                                                                                                                                                                                                 `ggggctcggagatgtgtataagagacagcGTAAATACAGGTTCACTTAGTGTGGACAATAAGAAGTTTTGGGCTACCGTAGAGTCCTCGGAGCATTCCTTCGAGGTTCCAATCTACGCTGAGACCCTAGACGAAGCTCTGGAGTTAGCCGAATGGCAAGACGTTCCGGCTGGCTTTGAGGTTACTCGTGTGCGTCCTTGTGTAGCACCGAAGTAATACGACTCACTATTAGGGAAGACTCCCTCTGAGAAACCAAACGAAACCTAAAGGAGATTAACATTATGGCTAAGAAGATTTTCAcc                                                               <  1:69004/273‑1 (MQ=255)`  
                                                                                                                                                                                                                                                   `aTCGAGAAGTCAATGACTATGTCAAACGTAAATACAGGTTCACTGAGTGTGGACAATAAGAAGGTTTGAGCTACCGTAGAGTCCTCGGAGCATTCCTTCGAGGTTCCAATCTACGCTGAGACCCTAGACGAAGCTCTGGAGTTAGCCGAATGGCAATACGTTCCGGCTGGCTTTGAGGTTACTCGTGTGCGTCCTTGTGTAGCACCGAAGTAATACGACTCACTATTAGGGAAGACTCCCTCTGAGAAACCAAACGAAACCTAAAGGAGATTAACATTATGGCTAAGAAGATTTTCACctc                                                             <  1:548402/301‑1 (MQ=255)`  
                                                                                                                                                                                                                                                          `ggTCAATGACTATGTCAAACGTAAATACAGGTTCACTTAGTGTGGACAATAAGAAGTTTTGGGCTACCGTAGAGTCCTCGGAGCATTCCTTCGAGGTTCCAATCTACGCTGAGACCCTAGACGAAGCTCTGGAGTTAGCCGAATGGCAATACGTTCCGGCTGGCTTTGAGGTTACTCGTGTGCGTCCTTGTGTAGCACCGAAGTAATACGACTCACTATTAGGGAAGACTCCCTCTGAGAAACCAAACGAAACCTAAAGGAGATTAACATTATGGCTAAGAAGATTTTCACCTCTGCGCTg                                                      <  1:795087/301‑1 (MQ=255)`  
                                                                                                                                                                                                                                                           `gTCAATGACTATGTCAAACGTAAATACAGGTTCACTTAGTGTGGACAATAAGAAGTTTTGGGCTACCGTAGAGTCCTCGGAGCATTCCTTCGAGGTTCCAATCTACGCTGAGACCCTAGACGAAGCTCTGGAGTTAGCCGAATGGCAATACGTTCCGGCTGGCTTTGAGGTTACTCGTGTGCGTCCTTGTGTAGCACCGAAGTAATACGACTCACTATTAGGGAAGACTCCCTCTGAGAAACCAAACGAAACCTAAAGGAGATTAACATTATGGCTAAGAAGATTTTctgtctcttatcca                                                     >  1:573100/1‑288 (MQ=255)`  
                                                                                                                                                                                                                                                           `gTCAATGACTATGTCAAACGTAAATACAGGTTCACTTAGTGTGGACAATAAGAAGTTTTGGGCTACCGTAGAGTCCTCGGAGCATTCCTTCGAGGTTCCAATCTACGCTGAGACCCTAGACGAAGCTCTGGAGTTAGCCGAATGGCAATACGTTCCGGCTGGCTTTGAGGTTACTCGTGTGCGTCCTTGTGTAGCACCGAAGTAATACGACTCACTATTAGGGAAGACTCCCTCTGAGAAACCAAACGAAACCTAAAGGAGATTAACATTATGGCTAAGAAGATTTTCACCTCTGCGCTgg                                                     >  1:288758/1‑301 (MQ=255)`  
                                                                                                                                                                                                                                                                `tGACTATGTCAAACGTAAATACAGGTTCACTTAGTGTGGACAATAAGAAGTTTTGGGCTACCGTAGAGTCCTCGGAGCATTCCTTCGAGGTTACAATCTACGCTGAGACCCTAGACGAAGCTCTGGAGTTAGCCGAATGGCAATACGTTCCGGCTGGCTTTGAGGTTACTCGTGTGCGTCCTTGTGTAGCACCGAAGTAATACGACTCACTATTAGGGAAGACTCCCTCTGAGAAACCAAACGAAACCTAAAGGAGATTAACATTATGGCTAAGAAGATTTTCACCTCTGCGCTTGGTAcc                                                >  1:816323/1‑301 (MQ=255)`  
                                                                                                                                                                                                                                                                       `gtcggagatgtgtataagagacagtAGTGGGGACAATAAGAAGTGTTGGGCTACCGTAGAGTCCTCGGAGCATTCCTTCGAGGTTCCAATCTACGCAGAGACCCTAGACGAAGCTCTGGAGTTAGCCGAATGGCAATACGTTCCGGCTGGCTTTGAGGTTACTCGTGTGCGTCCTTGTGTAGCACCGAAGTAATACGACTCACTATTAGGGAAGACTCCCTCTGAGAAACCAAACGAAACCTAAAGGAGATTAACATTATGGCTAAGAAGATTTTCACCTCTGCGCTGGGTACCGCTGAAc                                         <  1:170364/277‑1 (MQ=255)`  
                                                                                                                                                                                                                                                                       `gTCAAACGTAAATACAGGTTCACTTAGTGTGGACAATAAGAAGTTTTGGGCTACCGTAGAGTCCTCGGAGCATTCCTTCGAGGTTCCAATCTACGCTGAGACCCTAGACGAAGCTCTGGAGTTAGCCGAATGGCAATACGTTCCGGCTGGCTTTGAGGTTACTCGTGTGCGTCCTTGTGTAGCACCGCAGTAATACGACTCACTATTAGGGAAGACTCCCTCTGAGAACCCAAACGAAACCTAAATGAGATTAACATTATGGCTAAGAAGATTTTCACCTCTGCGCTGGGTACCGCTGAAc                                         >  1:67665/1‑301 (MQ=255)`  
                                                                                                                                                                                                                                                                        `tCAAACGTAAATACAGGTTCACTTAGTGTGGACAATAAGAAGTTTTGGGCTACCGTAGAGTCCTCGGAGCATTCCTTCGAGGTTCCAATCTACGCTGAGACCCTAGACGAAGCTCTGGAGTTAGCCGAATGGCAATACGTTCCGGCTGGCTTTGAGGTTACTCGTGTGCGTCCTTGTGTAGCACCGAAGTAATACGACTCACTATTAGGGAAGACTCCCTCTGAGAAACCAAACGAAACCTAAAGGAGATTAACATTATGGCTAAGAAGATTTTCACCTCTGCGCTGGGTACCGCTGAAcc                                        <  1:516832/301‑1 (MQ=255)`  
                                                                                                                                                                                                                                                                        `tCAAACGTAAATACAGGTTCACTTAGTGTGGACAATAAGAAGTTTTGGGCTACCGTAGAGTCCTCGGAGCATTCCTTCGAGGTTCCAATCTACGCTGAGACCCTAGACGAAGCTCTGGAGTTAGCCGAATGGCAATACGTTCCGGCTGGCTTTGAGGTTACTCGTGTGCGTCCTTGTGTAGCACCGAAGTAATACGACTCACTATTAGGGAAGACTCCCTCTGAGAAACCAAACGAAACCTAAAGGAGATTAACATTATGGCTAAGAAGATTTTCACCTCTGCGCTGGGTACCGCTGAAcc                                        <  1:140374/301‑1 (MQ=255)`  
                                                                                                                                                                                                                                                                           `aaCGTAAATACAGGTTCACTTAGTGTGGACAATAAGAAGTTTTGGGCTACCGTAGAGTCCTCGGAGCATTCCTTCGAGGTTCCAATCTACGCTGAGACCCTAGACGAAGCTCTGGAGTTAGCCGAATGGCAATACGTTCCGGCTGGCTTTGAGGTTACTCGTGTGCGTACTTGTGTAGCACCGAAGTAATACGACTCACTATTAGTGAAGACTCCCTCTGAGAAACCAAACGAAACCTAAAGGAGATTAACATTATGGCTAAGAAGATTTTCACCTCTGCGCTGGGTACCGCTGACCCTTa                                     >  1:245151/1‑301 (MQ=255)`  
                                                                                                                                                                                                                                                                             `cGTAAATACAGGTTCACTTAGTGTGGACAATAAGAAGTTTTGGGCTACCGTAGAGTCCTCGGAGCATTCCTTCGAGGTTCCAATCTACGCTGAGACCCTAGACGAAGCTCTGGAGTTAGCCGAATGGCAATACGTTCCGGCTGGCTTTGAGGTTACTCGTGTGCGTCCTTGTGTAGCACCGAAGTAATACGACTCACTATTAGGGAAGACTCCCTCTGAGAAACCAAACGAAACCTAAAGGAGATTAACATTATGGCTAAGAAGATTTTCACCTCTGCGCTGGGTACCGCTGAACCTTACg                                   >  1:345418/1‑301 (MQ=255)`  
                                                                                                                                                                                                                                                                              `gTAAATACAGGTTAACTGAGTGTGGACAATAAGAAGTTTTGGGCTACCGTAGAGTCCTCGGAGCATTCCTTCGAGGTTCCAATCTACGCTGAGACCCTAGACGAAGCTCTGGAGTTAGCCGAATGGCAATACGTTCCGGCTGGCTTTGAGGTTACTCGTGTGCGTCCTTGTGTTGCACCGAAGTAATACGACTCACTATTAGGGAAGACTCCCTCTGAGAAACCAAACGAAACCGAAAGGAGATTAACATTATGGCTAAGAAGAGTTTCACCTCTGCGCTGGGTACCGCTGAACCTTACGc                                  <  1:132841/301‑1 (MQ=255)`  
                                                                                                                                                                                                                                                                                         `ttCACTTAGTGTGGACAATAAGAAGTTTTGGGCTACCGTAGAGTCCTCGGAGCATTCCTTCGAGGTTCCAATCTACGCTGAGACCCTAGACGAAGCTCTGGAGTTAGCCGAATGGCAATACGTTCCGGATGGCTTTGAGGTTACTCGTGTGCGTCCTTGTGTAGCACCGAAGTAATACGACTCACTATTAGGGAAGACTCCCTCTGAGAACCCAAACGAAACCTAAAGGAGATTAACATTATGGCTAAGAAGATTTTCACCTCTGCGCTGGGTACCGCTGAACCTTACGCTTACATctgtt                       >  1:781916/1‑297 (MQ=255)`  
                                                                                                                                                                                                                                                                                            `aCTTAGTGTGGACAATAAGAAGTTTTGGGCTACCGTAGAGTCCTCGGAGCATTCCTTCGAGGTTCCAATCTACGCTGAGACCCTAGACGAAGCTCTGGAGTTAGCCGAATGGCAATACGTTCCGGCTGGCTTTGAGGTTACTCGTGTGCGTCCTTGTGTAGCACCGAAGTAATACGACTCACTATTAGGGAAGACTCCCTCTGAGAAACCAAACGAAACCTAAAGGAGATTAACATTATGGCTAAGAAGATTTTCACCTCTGCGCTGGGTACCGCTGAACCTTACACTTACATCGCCAAGc                    >  1:338166/1‑301 (MQ=255)`  
                                                                                                                                                                                                                                                                                                `aGTGTGGACAATAAGAAGTTTTGGGCTACCGTAGAGTCCTCGGAGCATTCCTTCGAGGTTCCAATCTACGCTGAGACCCTAGACGAAGCTCTGGAGTTAGCCGAATGGCAATACGTTCCGGCTGGCTTTGAGGTTACTCGTGTGCGTCCTTGTGTAGCACCGAAGTAATACGACTCCCTATTAGGGAAGACTCCCTCTGAGAAACCAAACGAAACCTACAGGCGATTAACATTATGGCTAAGAAGATTTTCACCTCTGCGCTGGGTACCGCTGAACCTTActgtctcttatacacatctcc                >  1:434038/1‑281 (MQ=255)`  
                                                                                                                                                                                                                                                                                                 `gtgtGGACAATAAGAAGTTTTGGGCTACCGTAGAGTCCTCGGAGCATTCCTTCGAGGTTCCAATCTACGCTGAGACCCTAGACGAAGCTCTGGAGTTAGCCGAATGGCAATACGTTCCGGCTGGCTTTGCGGTTCCTCGTGTGCGTCCTTGTGTAGCACCGAAGTAATACGACTCACTATTAGGGAAGACTCCCTCTGAGAAACCAAACGAAACCTAAAGGAGATTAACATTATGGCTAAGAAGATTTTCACCTCTGCGCTGGGTACCGCTGAACCTTACGCTTACATCTCCAAGCCAGAc               >  1:479177/1‑301 (MQ=255)`  
                                                                                                                                                                                                                                                                                                 `gtgtGGACAATAAGAAGTTTTGGGCTACCGTAGAGTCCTCGGAGCATTCCTTCGAGGTTCCAATCTACGCTGAGACCCTAGACGAAGCTCTGGAGTTAGCCGAATGGCAATACGTTCCGGCTGGCTTTGAGGTTACTCGTGTGCGTCCTTGTGTAGCACCGAAGTAATACGACTCACTATTAGGGAAGACTCCCTCTGAGCAACCAAACGAAATCTAAAGGAGAGTAACATTATGGCTAAGAAGATTTTCACCTCTGCGCTGGGTACCGCTGAACCTTACGCTTACATCGCCAAGCCGTAc               >  1:587486/1‑301 (MQ=255)`  
                                                                                                                                                                                                                                                                                                              `gaagTTTTGGGCTACCGTAGAGTCCTCGGAGCATTCCTTCGAGGTTCCAATCTACGCTGAGACCCTAGACGAAGCTCTGGAGTTAGCCGAATGGAAATACGTTCCGGCTGGCTTTGAGGTTACTCGTGTGCGTCCTTGTGTAGCACCGAAGTAATACGACTCACTATTAGGGAAGACTCCCTCTGAGAAACCAAACGAAACCTAAAGGAGATTAACATTATGGCTAAGAAGATTTTCACCTCTGCGCTGGGTACCGCTGAACCTTACGCTTACATCGCCAAGCCGGACTACGGCAACGAag  <  1:266408/301‑1 (MQ=255)`  

                                                                                                                                                                                                                                                                                                              `|`                                                                                                                                                                                                                                                                                                              
`TAAAGTACAGACTACGTGCTGGTAAGAAGTCAGAGTTAGCGTACTTAGAGAAAGACCTAGCGAAAGCAGACTTCTATAAAGAACTCTTTGAGAAACATAAGGATAAATGTTATGCATAACTTCAAGTCAACCCCACCTGCCGACAGCCTATCTGATGACTTCACATCTTGCTCAGAGTGGTGCCGAAAGATGTGGGAAGAGACATTCGACGATGCGTACATCAAGCTGTATGAAC‑TTTGGAAATCGAGAGGTCAATGACTATGTCAAACGTAAATACAGGTTCACTTAGTGTGGACAATAAGAAGTTTTGGGCTACCGTAGAGTCCTCGGAGCATTCCTTCGAGGTTCCAATCTACGCTGAGACCCTAGACGAAGCTCTGGAGTTAGCCGAATGGCAATACGTTCCGGCTGGCTTTGAGGTTACTCGTGTGCGTCCTTGTGTAGCACCGAAGTAATACGACTCACTATTAGGGAAGACTCCCTCTGAGAAACCAAACGAAACCTAAAGGAGATTAACATTATGGCTAAGAAGATTTTCACCTCTGCGCTGGGTACCGCTGAACCTTACGCTTACATCGCCAAGCCGGACTACGGCAACGAAG  >  NC_001604.1/8638‑9239`

  

Alignment Legend

Aligned base mismatch/match (shaded by quality score): `ATCG/ATCG` `< 3 ≤` `ATCG/ATCG` `< 17 ≤` `ATCG/ATCG` `< 28 ≤` `ATCG/ATCG` `< 37 ≤` `ATCG/ATCG` `< 38 ≤` `ATCG/ATCG`

Unaligned base: `atcg`    Masked matching base: `atcg`    Alignment gap: `‑`    Deleted base: `‑`

Reads not counted as support for junction

read\_name Not counted due to insufficient overlap past the breakpoint.

read\_name Not counted due to not crossing MOB target site duplication.
